# The morphology, molecular development and ecological function of pseudonectaries on *Nigella damascena* (Ranunculaceae) petals

**DOI:** 10.1038/s41467-020-15658-2

**Published:** 2020-04-14

**Authors:** Hong Liao, Xuehao Fu, Huiqi Zhao, Jie Cheng, Rui Zhang, Xu Yao, Xiaoshan Duan, Hongyan Shan, Hongzhi Kong

**Affiliations:** 1 State Key Laboratory of Systematic and Evolutionary Botany, CAS Center for Excellence in Molecular Plant Sciences, Institute of Botany, Chinese Academy of Sciences, 100093 Beijing, China; 2 University of Chinese Academy of Sciences, 100049 Beijing, China

**Keywords:** Evolutionary ecology, Evolutionary developmental biology, Plant evolution, Plant signalling

## Abstract

Pseudonectaries, or false nectaries, the glistening structures that resemble nectaries or nectar droplets but do not secrete nectar, show considerable diversity and play important roles in plant-animal interactions. The morphological nature, optical features, molecular underpinnings and ecological functions of pseudonectaries, however, remain largely unclear. Here, we show that pseudonectaries of *Nigella damascena* (Ranunculaceae) are tiny, regional protrusions covered by tightly arranged, non-secretory polygonal epidermal cells with flat, smooth and reflective surface, and are clearly visible even under ultraviolet light and bee vision. We also show that genes associated with cell division, chloroplast development and wax formation are preferably expressed in pseudonectaries. Specifically, *NidaYABBY5*, an abaxial gene with ectopic expression in pseudonectaries, is indispensable for pseudonectary development: knockdown of it led to complete losses of pseudonectaries. Notably, when flowers without pseudonectaries were arrayed beside those with pseudonectaries, clear differences were observed in the visiting frequency, probing time and visiting behavior of pollinators (i.e., honey bees), suggesting that pseudonectaries serve as both visual attractants and nectar guides.

## Abstract (web summary)

Interspecies interactions, including those between plants and pollinators, can involve deception. The authors characterize the molecular development of *Nigella damascena* pseudonectaries, and their adaptive function in attracting specific pollinators to concealed nectaries with visual cues.

## Introduction

Nectaries, the highly specialized glands or tissues that secrete nectar, are widespread in flowering plants (i.e., angiosperms) and play key roles in plant–animal interactions^1–4^. Nectaries can be floral or extrafloral and structural or nonstructural, depending on where they are located and how complex they are^5–7^. Floral nectaries with distinct structures are of particular interest to plant taxonomists and evolutionary biologists because they are usually tightly associated with pollination and because pollination is always the prerequisite of successful sexual reproduction^3,8–10^. In angiosperms, floral nectaries show considerable diversity in number, location (i.e., on sepals, petals, stamens, staminodes, carpels, or receptacle), and morphology (i.e., convex, concave, or disc- or cup-shaped), and are believed to be results of parallel or even convergent evolution^4,5,9,11^. Despite the diversity, it is widely accepted that the nectary has been one of the most influential key innovations that promoted the diversification of many plant lineages^7,12–14^. Meanwhile, it has been found that, to secrete nectar, nectary cells should be able to synthesize and transport sugar and other necessary components^10,15–17^; genes regulating the formation and proper functioning of nectaries, such as *CRABS CLAW* (*CRC*) of the *YABBY* family, *STYLISH1*(*STY1*), *STY2*, and *LATERAL ROOT PROMORDIUM* (*LRP*) of the *STY* family, and *SWEET9* of the *SWEET* family, therefore, are indispensable (at least in eudicots)^2,16,18–20^.

In addition to nectaries, many plants produce false nectaries, or pseudonectaries, that imitate or mimic nectaries or nectar droplets but do not secrete nectar^4,21–23^. Like nectaries, pseudonectaries also show considerable variation in size (from ~0.01 to ~0.5 cm), number (i.e., one, two, or many), location (i.e., on sepals, petals, stamens, or staminodes), color (i.e., green, yellowish, pink, or even black), and morphology (i.e., clavate, globular, hemispherical, convex, or patch- or cone-shaped)^22–33^. Yet, unlike nectaries, which have been investigated extensively in many aspects, pseudonectaries have not attracted sufficient attention until very recently^24,34^. Nevertheless, it has been shown that, acting as visual attractants or nectar guides, pseudonectaries of at least some plants play key roles in flower–animal interactions^25–27,35^. Pseudonectaries of *Lopezia* (Onagraceae) and *Pelargonium* (Geraniaceae), for example, can attract or guide their favorite pollinators (i.e., syrphids and *Megapalus capensis*, respectively) to proper positions and assist them to find the hidden nectary, suggestive of functional importance^25,27,33^. Pseudonectaries of carnivorous plants (e.g., *Cephalotus follicularis*) and deceptive flowers (e.g., *Ophrys muscifera*) also function to deceive the visiting insects^36,37^. Some studies also tried to understand the evolutionary histories of pseudonectaries^24^, yet the available data are still insufficient for a general picture. Meanwhile, due to the lack of suitable study systems, little is known about the morphological nature, optical features, developmental process, molecular underpinnings, and ecological functions of pseudonectaries.

**Fig. 1:**
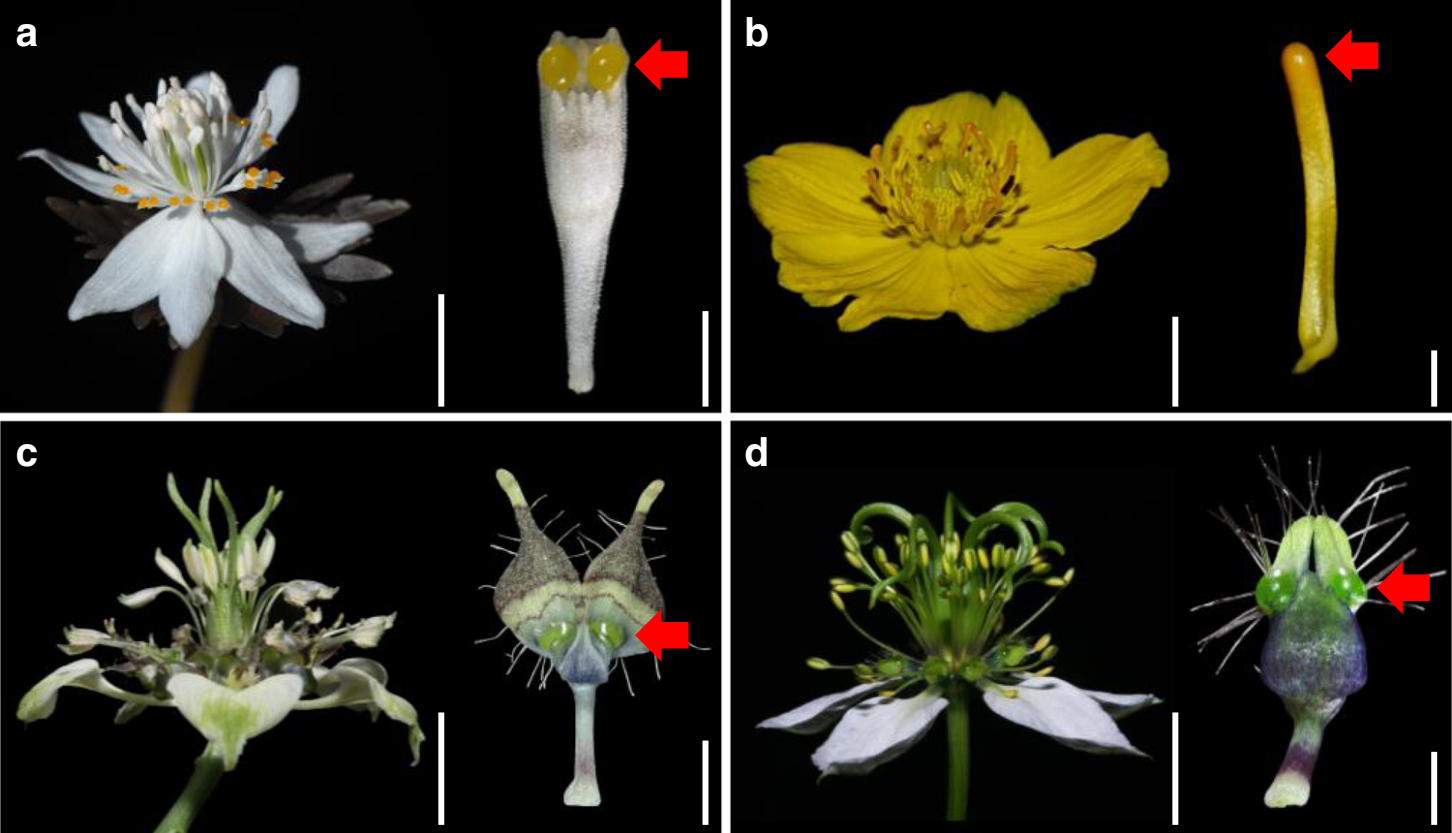
Flowers and their pseudonectary-bearing petals in representative species of the Ranunculaceae. **a** *Eranthis stellata*. **b** *Trollius buddae*. **c** *Nigella arvensis* ssp. *arvensis*. **d** *N. damascena*. Red arrows point to the pseudonectaries. Scale bars at the right side of flowers: 1 cm; scale bars at the right side of petals: 1 mm.

The family Ranunculaceae is an excellent system for the study of pseudonectary development and evolution, for three reasons. First, pseudonectaries, especially those that are located on petals, have been documented in at least four genera (i.e., *Nigella*, *Trollius*, *Eranthis*, and *Xanthorhiza*), and show considerable diversity in number, colour, morphology, and functions^24,32,38,39^ (Fig. 1). This provides an excellent opportunity for a comprehensive understanding of the generalities and peculiarities of pseudonectaries. Second, according to the recent results of ancestral character-state reconstruction, pseudonectaries of these taxa have been results of convergent evolution, followed by independent losses in a few cases^24,40^. This makes the family an excellent system for the study of the molecular bases of pseudonectary evolution. Third, one species of *Nigella*, *N. damascena*, has been developed into a model species, for which virus-induced gene silencing (VIGS) and many other functional technologies are applicable^24,41^. This suggests that the hypotheses related to pseudonectary development and evolution, if any, can be tested.

Here, by using *N. damascena* as a model, we investigate the morphological nature, optical features, developmental process, molecular bases, and ecological functions of pseudonectaries. We find that pseudonectaries are quite different from nectaries in morphological, anatomical, micromorphological, and functional properties, and that genes associated with cell division, chloroplast development, and wax formation are key to pseudonectary formation. In particular, an ortholog of an abaxial gene, *NidaYABBY5* (*NidaYAB5*), seems to be important for pseudonectary development: knockdown of it led to the complete losses of pseudonectaries and, therefore, significantly decreased the attractiveness of the petals. Our results not only provide a comprehensive portrait of pseudonectaries, but also clarify the differences between pseudonectaries and nectaries.

## Results

### Morphological, anatomical, and micromorphological features of pseudonectaries

**Fig. 2:**
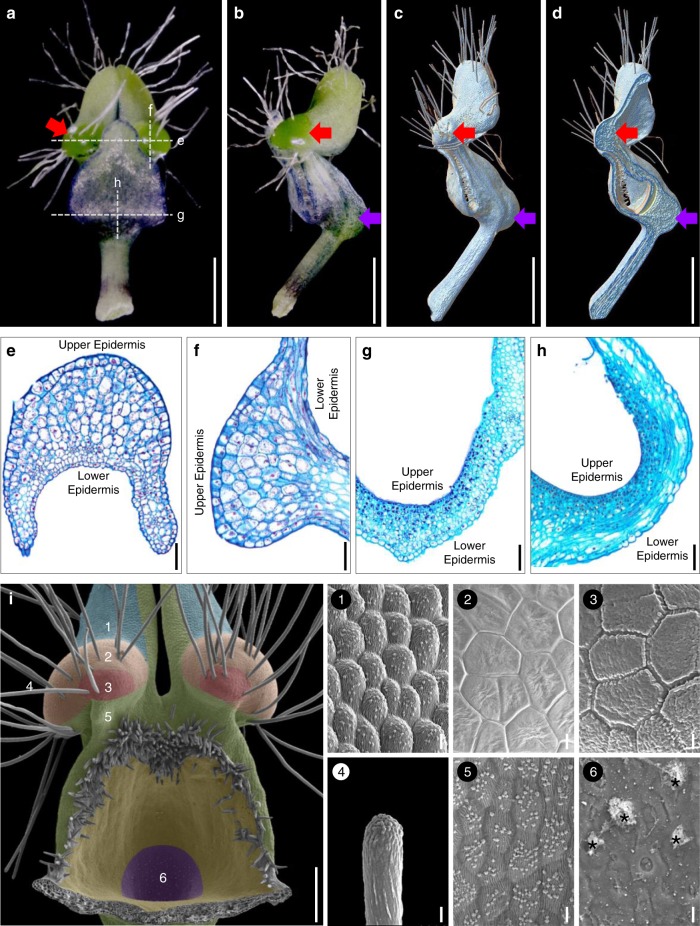
The pseudonectary morphology. Mature petals under stereomicroscope: adaxial (**a**) and lateral (**b**) views. **c–d** Mature petals under micro-CT. Red and purple arrows in **a**–**d** point to the pseudonectary and the nectary, respectively. Scale bars: 1 mm. Anatomy of the pseudonectary and nectary: transverse (**e**, **g**) and longitudinal (**f**, **h**) sections. Scale bars: 100 μm. **i** Micromorphology of the pseudonectary. The numbered regions (1–6) in different colors represent the distribution of different types of epidermal cells. Asterisks in (6) indicate residual nectar. Scale bars: **i**, 100 μm; 1–6, 10 μm. For **a**–**i** and 1–6, the experiments were repeated three times independently with similar results.

To understand the morphological nature of pseudonectaries, we first performed morphological, anatomical, and micromorphological studies. Under stereomicroscope, petals of *N. damascena* appear to be long-stalked, hair-bearing, doubly geniculated, and vertically bilabiate structures (Fig. 2a, b). Pseudonectaries, which are located at the distal geniculate bend of the lower petal lip, are hemispherical, emerald, glistening, and nectar droplet-like, and about 700 μm in diameter and 500 μm in height (Fig. 2a, b). Under X-ray microcomputed tomography (micro-CT), the pseudonectaries are obviously thicker than the other regions of the lower petal lip (Fig. 2c, d), suggesting that they were caused by regional thickening rather than simple surface curving. Under microscope, pseudonectaries are composed of 8–12 layers of irregularly arranged parenchyma cells covered by the tightly arranged epidermal cells (Fig. 2e, f). Both the parenchyma and epidermal cells are large in size and stained lightly and have large vacuoles and small nuclei (Fig. 2e, f). This, in fact, is also quite different from what we saw in nectary cells. In nectary tissues, the cells are small in size and stained darkly, suggestive of large nuclei and tiny or no vacuole (Fig. 2g, h). Under scanning electron microscopy (SEM), the features observable under stereomicroscope are even more obvious, and clear micromorphological differences can be seen between pseudonectaries and all other parts of the petal (Fig. 2i). Specifically, in addition to the long hairs, there are two types of polygonal cells (with smooth and grainy surfaces, respectively) that were exclusively found on pseudonectaries. Notably, however, it is these tightly arranged, nonsecretory polygonal epidermal cells with flat and smooth surface that can reflect light and make pseudonectaries shiny and attractive. Taken together, these results suggest that, as nectar droplet-like, nonsecretory protrusions with various exquisite morphological and optical modifications, pseudonectaries are quite different from nectaries and all other parts of the petal in many aspects.

### Cellular basis of pseudonectary development

**Fig. 3:**
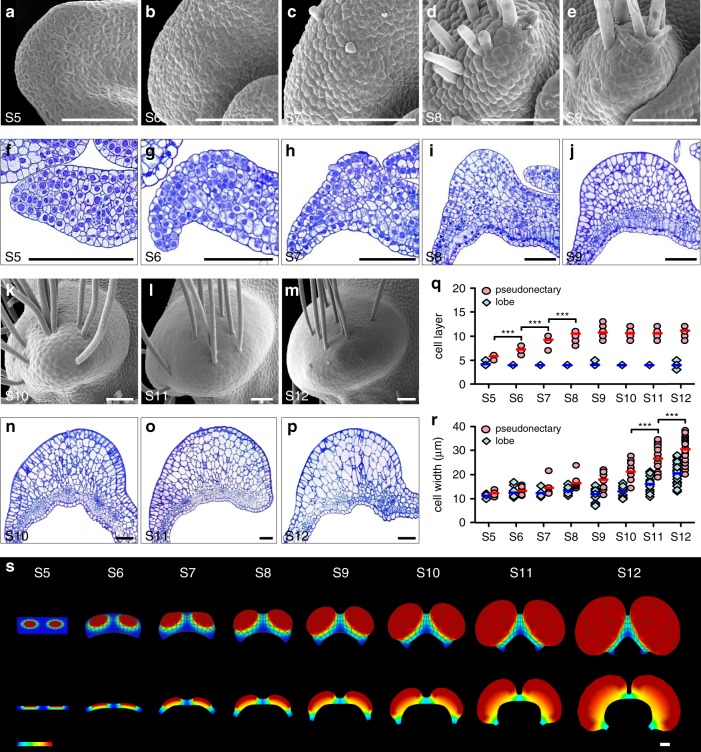
The pseudonectary development. Pseudonectaries at various developmental stages under SEM (**a**–**e**, **k**–**m**), with their corresponding transverse semi-thin sections being shown in (**f**–**j**, **n**–**p**). Scale bars: 100 μm. Comparisons of cell layer (**q**) and cell width (**r**) between the pseudonectary and lobe areas of petals at different stages. Bars indicate the mean values. The asterisks indicate the significant difference between adjacent stages by two-side Wilcoxon rank sum test (*P* < 0.001). The exact *P* values were listed in the Source Data. In **q**, *n* = 10 for S5, *n* = 19 for S6, *n* = 14 for S7, *n* = 16 for S8, *n* = 18 for S9, *n* = 10 for S10, *n* = 18 for S11, and *n* = 37 for S12; In **r**, *n* = 24 cells for S5, *n* = 42 cells for S6, *n* = 15 cells for S7, *n* = 33 cells for S8, *n* = 39 cells for S9, *n* = 30 cells for S10, *n* = 54 cells for S11, and *n* = 120 cells for S12. Source data for **q** and **r** are provided as a Source Data file. **s** Schematic of the pseudonectary development. The three-dimensional shapes and their corresponding transverse sections at S6, S7, S9, and S12 are shown, respectively. Heatmap is plotted denoting the relative growth rates. For **a**–**p**, the experiments were repeated four times independently with similar results.

To understand how pseudonectaries were made through development, we performed time-course micromorphological and anatomical studies. We found that pseudonectaries, as well as the long hairs on them, started to emerge at about the sixth stage (S6) of petal development^24^, likely due to active cell division underneath the epidermis (Fig. 3a–p). Then, during development, pseudonectaries became more and more protuberant and conspicuous, and eventually reached their final sizes at the twelfth stage (S12) of petal development (Fig. 3a–p). Notably, while both cell division and cell expansion have played key roles in pseudonectary development, their contributions are different: at the early stage of pseudonectary development (i.e., from S5 to S8 of petal development), the number of cell layers increased dramatically (Fig. 3q), suggestive of more active cell division; at the late stage of pseudonectary development (i.e., from S9 to S12 of petal development), the size of the cells increases more quickly (Fig. 3r), suggestive of more active cell expansion. Clearly, the ninth stage of petal development (S9) marks the transition between more active cell division and more active cell expansion during pseudonectary formation. Yet, it was the additive effect of cell division and cell expansion that led to the regional thickening and rapid formation of pseudonectaries (Fig. 3s).

### Genes involved in pseudonectary formation

**Fig. 4:**
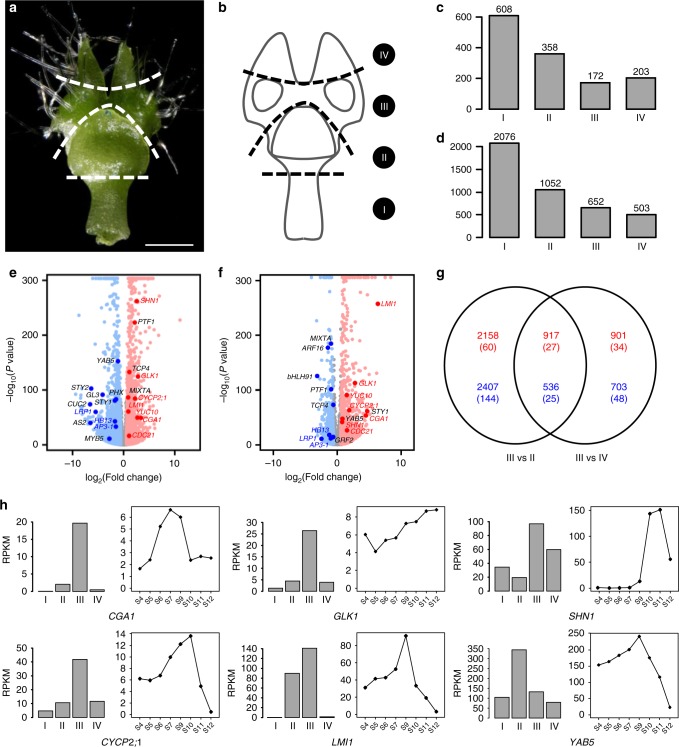
Identification of genes involved in pseudonectary formation. **a**, **b** Sampling strategy for RNA sequencing of the S9 petal. I, II, III, and IV represent the sampled four parts. Scale bar: 1 mm. For **a**, the experiments were repeated three times independently with similar results. Histograms showing the number of genes specifically (**c**) and preferably (**d**) expressed in each part. Volcano plots showing differentially expressed genes in Part III relative to Part II (**e**) and Part IV (**f**). The up- and downregulated genes are shown with red and blue dots, respectively, while the genes showing no significant expression changes are represented by gray dots. Representative genes homologous to the well-known regulators are highlighted with enlarged dots, with red and blue fronts standing for up- and downregulated genes, respectively, in both comparisons, while black front representing up- or downregulated genes in one comparison. **g** A Venn diagram showing the numbers of up- and downregulated genes (red and blue, respectively) in the two comparisons shown in **e** and **f**. A number of TFs are shown in brackets. **h** The spatiotemporal expression patterns of representative candidate genes.

To further understand the uniqueness of pseudonectaries, we divided the S9 petals into four parts (i.e., Parts I, II, III, and IV; Fig. 4a, b) and conducted RNA sequencing analyses. Of the 21,223 genes that are expressed in the petals of this stage, 172 and 652 are specifically and preferably expressed in the pseudonectary-containing Part III as compared with the other three parts (Fig. 4c, d; Supplementary Dataset 1), respectively, suggestive of the uniqueness of this part. Interestingly, of the genes that are preferably expressed in this part, there are homologs of the well-known regulators of photosynthetic apparatus and cell division, such as the *GOLDEN2-LIKE1* (*GLK1*) and *CYTOKININ-RESPONSIVE GATA FACTOR 1* (*CGA1*)^42,43^, and the gene ontology (GO) categories that were enriched include “photosynthesis”, “chlorophyll biosynthetic process”, and “response to cytokinin” (Supplementary Table 1). Genes involved in nectary development, such as orthologs of *STY1/2* and *LRP*^20^, however, are preferably expressed in the nectary-containing Part II (Supplementary Dataset 1), suggesting that pseudonectaries indeed have nothing to do with nectaries.

To identify the genes that are involved in pseudonectary formation, we also compared Part III with its neighboring regions (i.e., Parts II and IV) by using DESeq2^44^. We found that, compared with Part II, Part III has 3075 (including 87 transcription factor genes; hereafter called TFs) and 2943 (169 TFs) up- and down-regulated genes, respectively (Fig. 4e, g; Supplementary Dataset 1). Similarly, compared with Part IV, Part III has 1818 (61 TFs) and 1239 (73 TFs) up- and down-regulated genes, respectively (Fig. 4f, g; Supplementary Dataset 1), in which 917 (27 TFs) and 536 (25 TFs) were shared by the two comparisons (Fig. 4g; Supplementary Dataset 1). Of the genes that are up-regulated in both comparisons, there are homologs of the genes associated with cell division (e.g., *CGA1* and *CYCLIN P2;1*, *CYCP2;1*)^42,45^, chloroplast development (e.g., *GLK1*)^43^, wax formation (e.g., *SHINE1*, *SHN1*)^46^, and leaf morphogenesis (e.g., *LATE MERISTEM IDENTITY1*, *LMI1*)^47^ (Fig. 4h). Because these processes are required for the formation of pseudonectaries, it is very likely that they are key regulators of pseudonectary development.

### Importance of *NidaYAB5* in pseudonectary development

**Fig. 5:**
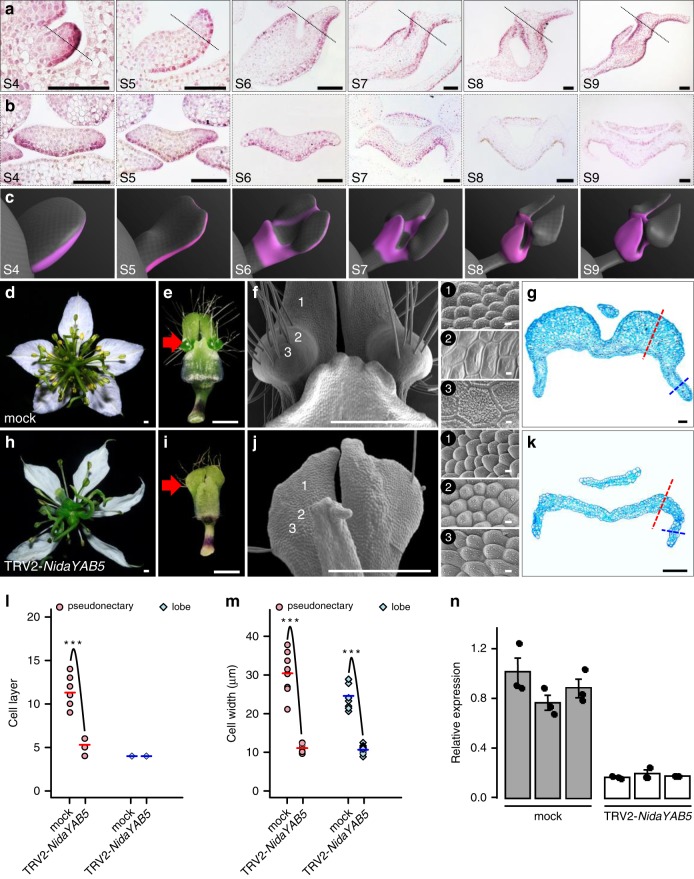
Expression and function of *NidaYAB5*. **a**, **b** The results of in situ hybridization of *NidaYAB5* in petals. Dashed lines in the longitudinal sections (**a**) indicate the positions at which the transverse sections (**b**) were made. Scale bars: 100 μm. For **a**, **b**, the experiments were repeated three times independently with similar results. **c** Virtual clay models showing the expression domains (pink) of *NidaYAB5* in petals. Phenotypes of the mock (**d**–**g**) and TRV2-*NidaYAB5*-treated flowers with strong phenotypic changes (**h**–**k**). The flowers and mature petals are shown in **d**, **h** and **e**, **i**, respectively. The micromorphology and anatomy of the pseudonectary regions are shown in **f**, **j** and **g**, **k**, respectively. 1–3 next to **f** and **j** indicate the corresponding regions on the surface of the mock and TRV2-*NidaYAB5*-treated petals. Red arrows point to the pseudonectary region, and dashed lines indicate the pseudonectary (red) and lobe (blue) areas at which the cell layer (**l**) and cell width (**m**) were recorded. Scale bars: **d**–**f**, **h**–**j**, 1 mm; 1–3, 10 μm; **g**, **k**, 100 μm. Comparisons of cell layer (**l**) and cell width (**m**) at the pseudonectary and lobe areas between mock and TRV2-*NidaYAB5*-treated flowers. Bars indicate the mean values. The asterisks indicate the significant difference by two-side Wilcoxon rank sum test (*P* value < 0.001) between samples. The exact *P* values were listed in the Source Data. Source data for **l** and **m** are provided as a Source Data file. **n** The results of qRT-PCR for *NidaYAB5* in petals of mock and TRV2-*NidaYAB5*-treated flowers. Each treatment includes three biological replicates. Error bars indicate the standard deviation (SD) of three technical replicates of each biological replicate. The measure of the center for the error bars is the mean value of three technical replicates of each biological replicate. Data are presented as mean values ± SD.

Of the genes that are upregulated in Part III, one (i.e., *NidaYAB5*; Fig. 4e, f, h) attracted our special attention because it is the ortholog of a known abaxial gene (i.e., *YAB5*)^24^ and because ectopic expression of adaxial/abaxial genes have been shown to play key roles in the formation of outgrowths on leaf-like structures^48–52^. To gain some insights into its function, we first performed detailed mRNA in situ hybridization studies (Fig. 5a–c). We found that, as expected, *NidaYAB5* was first expressed in the adaxial side of the upper lip and the abaxial side of the lower lip of the petals. Then, at S6, the signal of *NidaYAB5* also expanded to the places where pseudonectaries would be initiated, although the expression level was rather low. Thereafter, the expression of *NidaYAB5* in the developing pseudonectaries became stronger and stronger, and eventually reached the summit at S9 (Fig. 4h). Clearly, the ectopic expression of *NidaYAB5* strongly coincides with the formation of pseudonectaries.

To understand the function of *NidaYAB5*, we attempted to knock down its expression by using VIGS technique. Compared with the tobacco rattle virus (TRV2)-treated flowers (i.e., the mock) and TRV2-*NidaYAB5*-treated flowers with weak and moderate phenotypic changes, TRV2-*NidaYAB5*-treated flowers with strong phenotypic changes no longer produce pseudonectaries, whereas all other parts of the petals remain largely unaffected (Fig. 5d, e, h, i, n; Supplementary Fig. 1). Moreover, in the area where pseudonectaries were supposed to be, the polygonal epidermal cells with smooth and grainy surfaces were all transformed into conical cells, the highly specialized cell types that are widely distributed on the adaxial surface of the petal lobes (Fig. 5f, j). When sectioned, the number and size of the cells in the pseudonectary regions decreased dramatically, whereas the number of cells in the lobe regions were largely not affected (Fig. 5g, k–m). This confirms that *NidaYAB5* plays key roles in pseudonectary development.

### The contribution of pseudonectaries to pollination success

**Fig. 6:**
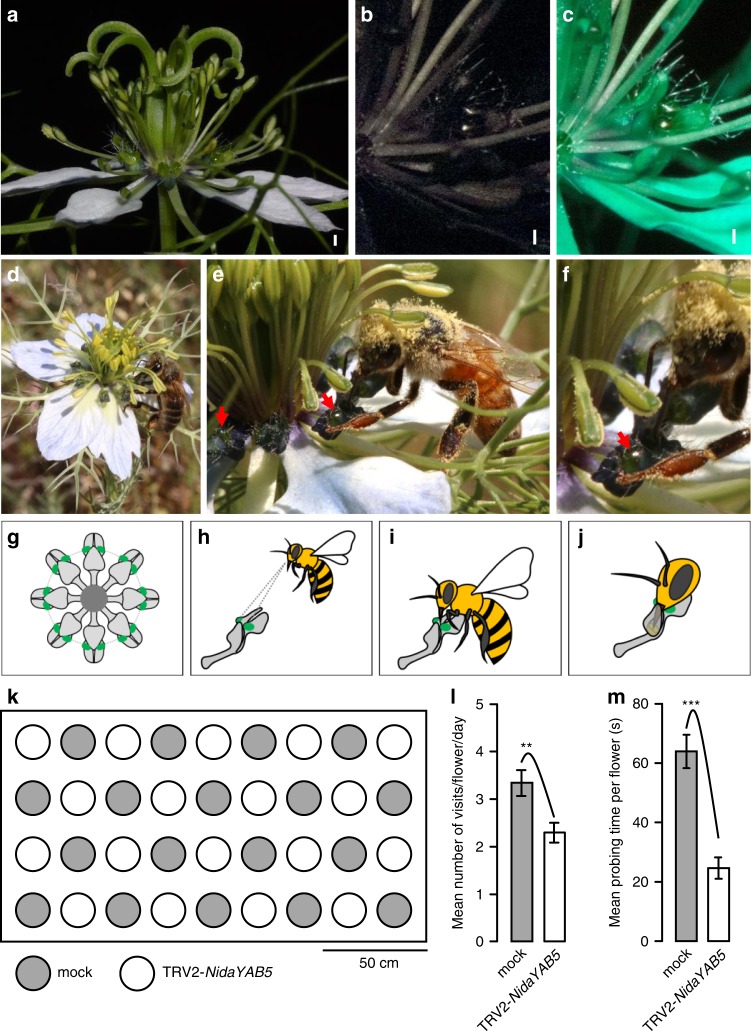
The optical properties and ecological function of pseudonectaries. A wild-type flower under visible light (**a**), ultraviolet light (**b**), and bee vision (**c**). Scale bars: 1 mm. **d**–**f** Photographs show honey bees (*Apis mellifera*) forging for nectar. Red arrows indicate the pseudonectaries. The pseudonectary in **e** is magnified and shown in **f**. **g** Petals and pseudonectaries in a wild-type flower, with all other floral organs being omitted. **h**–**j** Visitation of a petal by a honeybee. **k** Schematic layout of the mock and TRV2-*NidaYAB5*-treated flowers with strong phenotypic changes for pollination studies. Comparisons of the visiting frequency (**l**) and probing time (**m**) of honey bees, the most effective pollinators, on the mock and TRV2-*NidaYAB5*-treated flowers. The asterisks indicate the significant differences by two-side Wilcoxon rank sum test (*P* < 0.01 and *P* < 0.001, respectively) between samples. *p* = 0.006654 for **l** and *p* = 1.396e^−08^ for **m**. *n* = 18, both for mock and TRV2-*NidaYAB5*-treated flowers for four independent experiment days. Error bars indicate the standard error (SE) of the mean visiting frequency or probing time of 4 days. Data are presented as mean values ± SE. Source data for **l** and **m** are provided as a Source Data file.

Previous studies have proposed that pseudonectaries may function as nectar guides or visual attractants^4,21,22,27,53^. To test this hypothesis, we first examined the optical properties of pseudonectaries (Fig. 6a–c). We found that, under ultraviolet (UV) light, pseudonectaries are shiny and reflective, suggestive of UV reflection, whereas all other parts of the petals and the flower are dark black (Fig. 6b), suggestive of UV absorption. Under bee vision, the pseudonectaries are still shiny and reflective, while all other parts of the petals and the flower become dark green (Fig. 6c), suggesting that the pseudonectaries may be seen by bees. In addition, because of the formation of two quasi-perpendicular, geniculate bends on the lower petal lip, pseudonectaries became more conspicuous than any other parts of the flower. When all petals of a flower were considered together, the pseudonectaries form a concentric circle, in which the two from each petal mark the entrance of the nectary chamber (Fig. 6d–j).

To further understand the function of pseudonectaries, we performed pollination studies. We found that, consistent with previous studies^54^, the most frequent visitors and effective pollinators of *N. damascena* are honey bees (*Apis mellifera*), although the contribution of bumblebees (*Bombus lucorum*) and wasps (*Polistes dominulus*) were also substantial (about 10% of the recorded times of visitations). When flowers with and without pseudonectaries (i.e., mock flowers and the TRV2-*NidaYAB5*-treated flowers with strong phenotypic changes, respectively) were arrayed side by side (Fig. 6k), both types could attract honey bees. However, both the visiting frequency and probing time of honey bees decreased significantly in flowers without pseudonectaries than in those with pseudonectaries (Fig. 6l, m), suggestive of the differences in attractiveness. More interestingly, when a pollinator landed on a flower with pseudonectaries, it tried to check every petal in a clockwise or anticlockwise direction; when it landed on a flower without pseudonectaries, however, it usually flew away after brief tries. Taken together, these results tend to suggest that pseudonectaries can not only attract suitable pollinators but also mark the entrance of the nectar chamber, thereby guiding their visitation.

## Discussion

In this study, by conducting careful morphological, anatomical, and micromorphological studies, we uncovered the morphological nature of pseudonectaries. We found that pseudonectaries of *N. damascena* (and other species of *Nigella*) are protrusive, emerald, glistening, and nonsecretory structures that mimic nectar drops but do not produce and secrete nectar. We also found that under UV light and bee vision, pseudonectaries are shiny and reflective, whereas all other parts of the petals and the flower are black or dark green. This, together with the observation on many other plants^22,28,39,55,56^, suggests that protrusion, coloration, reflectivity, and being nonsecretory may be the most important features that characterize pseudonectaries. Specifically, being protrusive, colorful, and reflective makes pseudonectaries visible and attractive to specific pollinators (usually bees and flies), whereas being nonsecretory makes pseudonectaries functionally distinct to real nectaries.

Several scenarios have been proposed for the ecological functions of pseudonectaries. Based mainly on morphological observations, many authors believe that, by forming protrusive, colorful, and glistening structures that mimic nectaries, nectar droplets or even pollinators, pseudonectaries serve to optically attract pollinators^21,22,29,56^. Some other authors, however, insist that in addition to optical attraction, pseudonectaries can tell pollinators the position of hidden nectar or pollens, thereby guiding the visitation^25,27,31^. While these two scenarios are not mutually exclusive, there is a third viewpoint, which postulates that pseudonectaries function to distract undesirable visitors from rewards intended for pollinators^53^. In this study, we not only examined the optical features of pseudonectaries but also conducted controlled experiments. We found that pseudonectaries are indeed visible to pollinators (i.e., bees), and that pseudonectaries from all the petals of a flower form a concentric circle, in which the two from each petal mark the entrance of the nectary chamber. When flowers without pseudonectaries were arrayed beside the ones with pseudonectaries, clear differences were observed in the visiting frequency, probing time and visiting behavior of the pollinators. This suggests that pseudonectaries can not only optically attract pollinators but also help them find the hidden nectar, thereby guiding their visitation. The distraction scenario of pseudonectaries, however, cannot be supported or rejected in this study.

It is interesting that plants bearing pseudonectaries usually also produce real nectaries. If the functions of pseudonectaries are to attract pollinators and help them find the hidden nectar, why do plants hide their nectar? One explanation is that exposed nectar that can be easily foraged by both preferred and nonpreferred visitors and/or quickly dry off, thereby causing the waste of energy. The plants, therefore, have evolved various strategies to hide their nectar. Indeed, in most of the plants with floral pseudonectaries, nectar and real nectaries are very well hidden, either in the spurs or pockets of petals or in the tube formed by corolla^22^. However, in many of these cases, it would become difficult even for the preferred visitors and real pollinators to find and reach the nectar. The formation of pseudonectaries, hence, would be one of the best strategies to attract preferred visitors without losing the hidden nectar.

It is interesting that, by conducting extensive transcriptomic and functional studies, we identified the genes and networks that likely play key roles in the formation of the various aspects of pseudonectaries. Genes involved in cell division and cell expansion (e.g., *CGA1*, *CYCP2;1*, and *YUCCA10*), for example, seem to be required for the outgrowth of pseudonectaries, while those associated with chloroplast development (e.g., *GLK1*) and wax formation (e.g., *SHN1*) are indispensable for the formation of optical features^43,46^. Genes involved in nectary development, such as orthologs of *STY1/2* and *LRP*, however, are not required, suggesting that pseudonectaries indeed have nothing to do with nectaries. In addition, we found that the abaxial gene *NidaYAB5* is a key regulator of pseudonectary formation; knockdown of it not only led to complete losses of pseudonectaries but also eliminated all cell types associated with pseudonectories. It is possible that the gene was initially ectopically expressed on the adaxial surface to promote outgrowth but then controls all aspects of pseudonectary development by regulating its downstream genes. Notably, consistent with the widely accepted theory of leaf-like structure formation^51^, ectopic expression of *NidaYAB5* can explain the reason why pseudonectaries were formed in the adaxial side of the lower lip of the petal.

It is worth mentioning that in *Nigella*, pseudonectaries are actually a kind of new character originated during the evolution of the genus and that pseudonectaries of different species show very little variation in morphology, micromorphology, and developmental process^24^. Therefore, if ectopic expression of *YAB5* can explain the reason why pseudonectaries of one species are formed, it can explain the formation of pseudonectaries in other species of *Nigella*. In reality, however, it is still difficult to conclude that ectopic expression of *NidaYAB5* is the sole cause for the formation and origination of pseudonectaries in *Nigella* because changes in the expression pattern of a gene can be caused by many factors, such as alterations of the *cis*-regulatory elements of the gene or mutations of its upstream transcription factors^30,57^. In addition, previous studies have shown that regional cell division and/or cell expansion, as well as reactivation of the meristematic program, can also lead to the formation of a protrusion on the surface of lateral organs^58,59^, suggestive of the complexity of the problem. More in-depth studies, therefore, are required to uncover the mechanisms underlying the formation, development, and origination of pseudonectaries.

## Methods

### Plant materials and growth conditions

Seeds of *N. damascena*, purchased from B & T World Seeds (Paguignan, France), were sown in soil (vermiculite:nutrient soil = 2:1) and grown under conditions of 24 °C, 60% relative humidity, and a 12-h-light/12-h-dark photoperiod.

### Microscopy and histology

For stereomicroscope photographing, typical mature petals of *N. damascena* were dissected and photographed with a Nikon Model C-DSS230 stereomicroscope assembled with a Nikon digital camera DXM1200F (Nikon Instech Co. Ltd, Kawasaki, Japan). For micro-CT scanning, typical mature petals of *N. damascena* were mounted in a plastic box with wet absorbent paper paved at the bottom to prevent dehydration and were subject to take the high-resolution three-dimensional (3D) images (16-bit .tif format) by using a micro-CT scanner (Bruker Sky Scan 1172; Bruker Corp., Billerica, MA, USA). Three-dimensional reconstructions were then performed with NRECON v.1.6 to remove the noise and convert the slices into an 8-bit .bmp format. Regions of interest were further selected by using CTAN v.1.10 and saved as datasets. The datasets were then loaded into CTVOX v.2.2 for the manipulation of 3D surface-rendered models. The internal sections of interest within the petal model were explored with the cutting/clipping shape editor and were captured as screenshots for showing. For SEM, typical petals of *N. damascena* at different developmental stages were fixed in fresh FAA (3.7% formaldehyde, 5% acetic acid, and 50% ethanol), followed by dehydration in a graded water–ethanol series, and dried with a CO_2_ critical-point dryer. After being sputter-coated with gold, the dried petals were examined with a Hitachi S-4800 scanning electron microscope. For histological observation, paraffin and semi-thin section series were performed. For the former, the pretreatment, embedding, and sectioning of petals were carried out as described^24^ but sections were stained with safranine and fast green. For the latter, petals were fixed in 2.5% glutaraldehyde (pH 7.2) and embedded in Spurr resin. Serial sections were prepared on an ultramicrotome Leica EM UC7 and stained with 0.33% toluidine blue. The sections were photographed with a Leica DM5000B light microscope. The cell layer of the pseudonectary and lobe areas in the median transverse semi-thin sections was counted directly, and the width of three representative cells in each area was measured using tpsUtil and tpsDIG2^60^. Significance evaluation (*P* value) between adjacent stages was evaluated using the Wilcoxon rank sum test.

### Computational modeling

The pictures in Fig. 3s were generated by using GFtbox (http://sourceforge.net/projects/gftbox) with two coordinated networks: the Polarity Regulatory Network and Growth Rates Regulatory Network^61,62^. The initial state was a mesh which consists of 36,000 finite elements with a grid showing the deformation. The elastic growth rate across the whole tissue is isotropic (Kpar = Kpar2 = Kper), and is higher in the region where pesudonectaries will be formed. Code is available upon request.

### RNA sequencing analyses

The four parts of S9 petals, each with three biological replicates, were subjected to total RNA extraction using the SV Total RNA Isolation System (Promega). A total of 12 libraries were constructed independently for single-end 100-bp-long reads sequencing on Illumina HiSeq2000. The clean reads of 12 samples were separately mapped to the reference transcriptome of *N. damascena*^41^ and the Reads Per Kilo bases per Million reads (RPKM) values were calculated by RSEM^63^. The quality of all the 12 transcriptomes was reflected by reads mapping rates (83.19–84.57%) and Pearson correlation coefficients between triplicates (0.99–1.00). The genes showing RPKM ≥ 1 in at least one part are defined as expressed. Meantime, a gene is considered as specifically expressed if its RPKM ≥ 1.0 in a specific part but <1.0 in other parts. A gene is regarded as preferably expressed if its RPKM in a specific part is at least 1.5-fold higher than those in any other parts. For GO analysis, the protein sequences of genes that are preferably expressed in Part III were BLAST against the *Arabidopsis thaliana* non-redundant protein database with an *E* value cut-off <1e^−10^^64^. The GO terms of each gene were determined according to those of its best hit in *A. thaliana*. The GO enrichment was carried out using the agriGO program with false discovery rate ≤ 0.05^65^. Differential expression analyses between Part III and its neighboring areas were performed using DESeq2 package in R^44^, Wald test, Benjamini and Hochberg’s correction. The cut-off value of fold change and *P* value were 1.5 and 0.05, respectively. The *P* values were adjusted using the Benjamini–Hochberg procedure^66^.

### Expression and functional studies

mRNA in situ hybridization was used to investigate the expression patterns of *NidaYAB5* following the procedure as described^24^. VIGS was applied to study the function of *NidaYAB5*. The same gene fragment used for the in situ hybridization was amplified and introduced into the TRV2-based pYL156 vector, which was electroporated into *Agrobacterium tumefaciens* strain GV3101. The detailed procedure of construct transformation and plant treatment followed the previous study^41^. A total of three rounds of treatments were conducted with TRV2-*NidaYAB5* (Supplementary Table 3). Meanwhile, parallel treatments with the empty TRV2 vector were also performed as a negative control (mock). The morphology, micromorphology, and histology of flowers with visible phenotypic changes were investigated as described above. The efficiency of the silencing was checked by quantitative reverse-transcription PCR (qRT-PCR) as described^41^. The primers used for vector construction and qRT-PCR were listed in Supplementary Table 2.

### Examination of optical properties

The same blooming flower under visible light, UV light, and bee vision was photographed by a converted digital camera SONY NEX-7 with BG39 filter, BG39 + ZWB3 filters, and BG39 + ZWB1 filters, respectively.

### Pollination studies

Pollination studies were carried out in an open area of the Institute of Apicultural Research, Chinese Academy of Agricultural Sciences from 9:00 a.m. to 5:00 p.m. in four sunny and calm days of July, 2017. Visitors being able to successfully transfer pollens from pollen sac to stigma were regarded as pollinators, while those that suck nectar but do not transfer pollens were regarded as nectar robbers. The most effective pollinators are the pollinators that have the highest frequency of visitation and longest time of probing. For the experiment, flowers with and without pseudonectaries (i.e., 18 mock flowers and 18 TRV2-*NidaYAB5*-treated flowers with strong phenotypic changes) were arrayed side by side (Fig. 6k). For each kind of potential pollinators, both the visiting frequency and probing time were recorded. After filtering inefficient data (i.e., visitation of nectar robbers), significance evaluation of the two pollination parameters (*P* value) were determined by using nonparametric Wilcoxon signed rank test.

### Reporting summary

Further information on research design is available in the Nature Research Reporting Summary linked to this article.

## Supplementary information

Peer Review

Supplementary Information

Reporting summary

Description of Additional Supplementary Files

Supplementary Dataset 1

## Data Availability

The RNA-seq data have been deposited in NCBI Short Read Archive with accession number PRJNA611670. All other data supporting the findings of this study are available within the paper and its Supplementary Information files. The source data underlying Figs. 3q, r, 5l, m, and 6l, m and Supplementary Fig. 1j are provided as a Source Data file.

## Source Data

Source Data

## Code availability

Codes for computational modeling, differential expression analyses are available upon request.
