# Author Correction: The morphology, molecular development and ecological function of pseudonectaries on *Nigella damascena* (Ranunculaceae) petals

**DOI:** 10.1038/s41467-020-16194-9

**Published:** 2020-05-06

**Authors:** Hong Liao, Xuehao Fu, Huiqi Zhao, Jie Cheng, Rui Zhang, Xu Yao, Xiaoshan Duan, Hongyan Shan, Hongzhi Kong

**Affiliations:** 1 State Key Laboratory of Systematic and Evolutionary Botany, CAS Center for Excellence in Molecular Plant Sciences, Institute of Botany, Chinese Academy of Sciences, 100093 Beijing, China; 2 University of Chinese Academy of Sciences, 100049 Beijing, China

**Keywords:** Evolutionary ecology, Evolutionary developmental biology, Plant evolution, Plant signalling

Correction to: *Nature Communications* 10.1038/s41467-020-15658-2, published online 14 April 2020.

In the original version of the Article, there were typographical errors in the Abstract and Discussion sections.

In the original Abstract, the gene ‘*NidaYABB5’* was missing a Y and should have been *NidaYABBY5*.

In the first paragraph of the Discussion, the sentence ‘Specifically, being protrusive, colorful, and reflective make pseudonectaries…’ should have read: ‘Specifically, being protrusive, colorful, and reflective makes pseudonectaries…’.

In the fourth paragraph of the Discussion, the sentence ‘Genes involved in nectar development, such as orthologs *STY1/2* and *LRP*…’ should have read: ‘Genes involved in nectary development, such as orthologs of *STY1/2* and *LRP*…’.

In the fourth paragraph of the Discussion, the sentence ‘…ectopic expression of *NidaYAB5* can explain the reason why pseudonectaries was formed in the adaxial side of the lower lip of the petal’ should have read: ‘…ectopic expression of *NidaYAB5* can explain the reason why pseudonectaries were formed in the adaxial side of the lower lip of the petal’.

This has now been corrected in the PDF and HTML versions of the Article.

