# Supplementary material for: The morphology, molecular development and ecological function of pseudonectaries on Nigella damascena (Ranunculaceae) petals
Source: Nat Commun. 2020 Apr 14;11:1777. doi: 10.1038/s41467-020-15658-2 (PMC7156421; doi:10.1038/s41467-020-15658-2)
Supplement: Supplementary file 2 — Supplementary Information [file 41467_2020_15658_MOESM2_ESM.pdf]

SUPPLEMENTARY FIGURES AND TABLES FOR

The morphology, molecular development and ecological function of pseudonectaries on *Nigella damascena* (Ranunculaceae) petals

Liao et al.

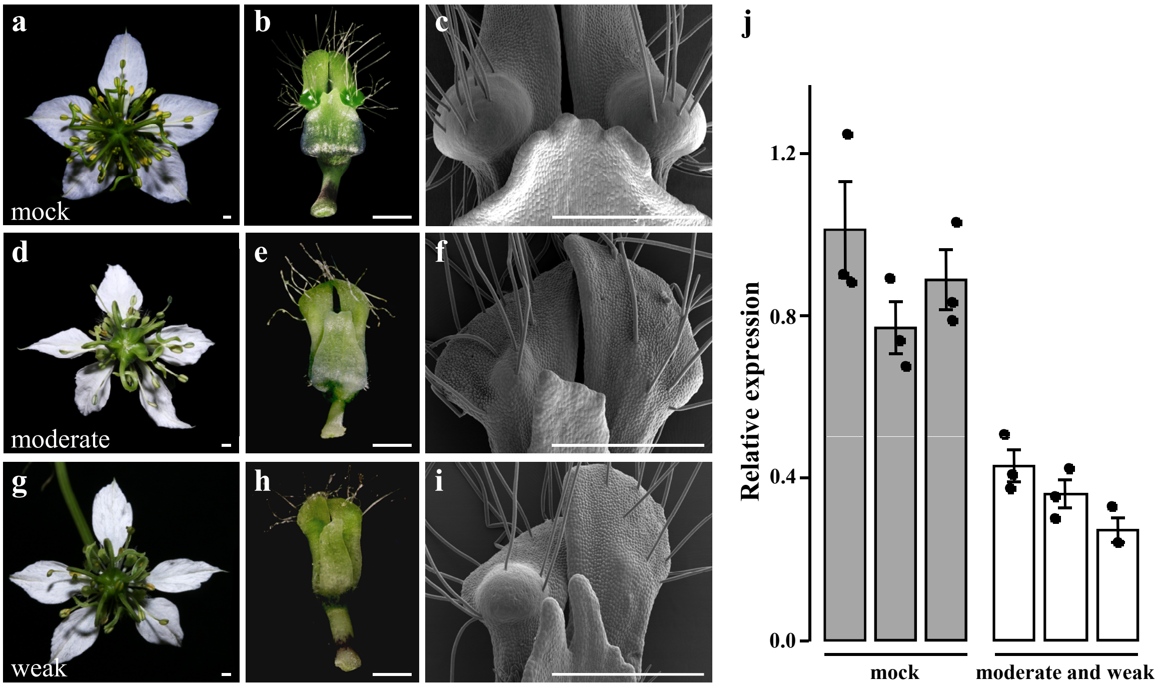

Supplementary Figure 1. Phenotypes of VIGS-treated flowers. The flowers and mature petals of the mock (**a-c**) and TRV2-*NidaYAB5*-treated flowers with moderate (**d-f**) and weak (**g-i**) phenotypic changes are shown in (**a**,**d**,**g**) and (**b**,**e**,**h**), respectively. The micromorphology of the pseudonectary regions are shown in (**c**,**f**,**i**). Scale bars: 1mm. Results of qRT-PCR for *NidaYAB5* in petals of mock and TRV2-*NidaYAB5*-treated flowers (**j**). Each treatment includes three biological replicates. Error bars indicate the standard deviation (SD) of three technical replicates of each biological replicate. The measure of the centre for the error bars is the mean value of three technical replicates of each biological replicate. Data are presented as mean values +/- SD. Source data for (**j**) is provided as a Source Data file.

Supplementary Table 1 Enriched GO terms of preferably expressed genes in Part III of S9 petals.

GO terms	Description (Biological Process)	No. of input	No. of Background	*P* value	FDR
GO:0015979	photosynthesis	78	256	3.3E-55	9.3E-52
GO:0019684	photosynthesis, light reaction	53	155	1.7E-41	2.4E-38
GO:0009768	photosynthesis, light harvesting in photosystem I	25	30	2.2E-34	2.1E-31
GO:0044765	generation of precursor metabolites and energy	62	353	1E-29	7.3E-27
GO:0009765	photosynthesis, light harvesting	26	50	3.2E-27	1.8E-24
GO:0018298	protein-chromophore linkage	24	52	1.2E-23	5.7E-21
GO:0009767	photosynthetic electron transport chain	22	70	1.7E-17	6.9E-15
GO:0055114	oxidation-reduction process	93	1238	5.1E-16	1.8E-13
GO:0022900	electron transport chain	26	137	1.6E-14	5.1E-12
GO:0009416	response to light stimulus	54	657	9.4E-12	2.7E-09
GO:0009773	photosynthetic electron transport in photosystem I	10	19	2E-11	5.2E-09
GO:0009314	response to radiation	54	679	3.2E-11	7.6E-09
GO:0044710	single-organism metabolic process	159	3215	3.9E-10	8.7E-08
GO:0009644	response to high light intensity	14	89	1.8E-07	3.6E-05
GO:0009642	response to light intensity	18	148	2E-07	3.8E-05
GO:0042548	regulation of photosynthesis, light reaction	8	30	1.2E-06	0.0002
GO:0043467	regulation of generation of precursor metabolites and energy	8	30	1.2E-06	0.0002
GO:0010109	regulation of photosynthesis	9	47	5E-06	0.0008
GO:0010114	response to red light	9	50	8.6E-06	0.0013
GO:0009628	response to abiotic stimulus	84	1796	1.1E-05	0.0015
GO:0009145	purine nucleoside triphosphate biosynthetic process	8	41	1.4E-05	0.002
GO:0006754	ATP biosynthetic process	7	32	2.3E-05	0.0027
GO:0015985	energy coupled proton transport, down electrochemical gradient	7	32	2.3E-05	0.0027
GO:0015986	ATP synthesis coupled proton transport	7	32	2.3E-05	0.0027
GO:0010218	response to far red light	8	44	2.5E-05	0.0029
GO:0044711	single-organism biosynthetic process	68	1432	3.7E-05	0.0041
GO:0042181	ketone biosynthetic process	8	47	4.1E-05	0.0044
GO:0009142	nucleoside triphosphate biosynthetic process	8	48	4.8E-05	0.0049
GO:0046148	pigment biosynthetic process	15	162	5.3E-05	0.0052
GO:0009645	response to low light intensity stimulus	5	17	7.5E-05	0.0072
GO:0009206	purine ribonucleoside triphosphate biosynthetic process	7	40	0.0001	0.0095

GO:0009735	response to cytokinin	18	233	0.00011	0.0098
GO:0042440	pigment metabolic process	16	193	0.00011	0.0098
GO:0009081	branched-chain amino acid metabolic process	5	19	0.00013	0.011
GO:0010411	xyloglucan metabolic process	6	30	0.00015	0.012
GO:0009201	ribonucleoside triphosphate biosynthetic process	7	44	0.00019	0.015
GO:0043155	negative regulation of photosynthesis, light reaction	5	21	0.00022	0.017
GO:0010410	hemicellulose metabolic process	8	59	0.00022	0.017
GO:0010205	photoinhibition	5	21	0.00022	0.017
GO:0015995	chlorophyll biosynthetic process	8	63	0.00034	0.024
GO:0044550	secondary metabolite biosynthetic process	14	173	0.00037	0.026
GO:0044281	small molecule metabolic process	65	1482	0.00042	0.028
GO:1905156	negative regulation of photosynthesis	5	24	0.00044	0.029
GO:1901659	glycosyl compound biosynthetic process	9	83	0.00049	0.032
GO:0009694	jasmonic acid metabolic process	7	52	0.00055	0.035
GO:0010207	photosystem II assembly	5	26	0.00065	0.04
GO:0015994	chlorophyll metabolic process	9	87	0.0007	0.041
GO:0010817	regulation of hormone levels	16	227	0.00069	0.041
GO:0009637	response to blue light	8	71	0.00077	0.045

Supplementary Table 2 Primers used in this study.

Purpose	Primer name	Primer sequence (5’→3’)
**VIGS**		
NidaYAB5	YAB5_V_F	CAGGTACCGTAACTGTACGTTGTGGGC
NidaYAB5	YAB5_V_R	TACTCGAGGCCTTAATTCTCTGGATTTC
**qRT-PCR**		
NidaYAB5	YAB5_qRT_F	CACTGACCCCATTCCCACTC
NidaYAB5	YAB5_qRT_R	GGCTTGTAACAACTTTCACCTC
ACTIN	ACTIN_qRT_F	CCGTTCTGCTGTGGTTGTG
ACTIN	ACTIN_qRT_R	GTGTTGGACTCTGGTGATGG

Supplementary Table 3 Information on VIGS experiments.

Batch	Plants			Flowers		
	**Treated**	**Survived**	**Recorded**	**Strong**[a]	**Moderate**[b]	**Weak**[c]
1	80	32	5	4	10	2
2	104	40	40	5	16	0
3	96	76	76	69	106	14
Total	280	148	121	78	132	16

[a]Strong: No pseudonectaries are formed on any of the petals in a flower.

[b]Moderate: More than 50% pseudonectaries cannot be formed or show obvious defects.

[c]Weak: More than 50% pseudonectaries show no phenotypic changes.
