# Supplementary material for: The morphology, molecular development and ecological function of pseudonectaries on Nigella damascena (Ranunculaceae) petals
Source: Nat Commun. 2020 Apr 14;11:1777. doi: 10.1038/s41467-020-15658-2 (PMC7156421; doi:10.1038/s41467-020-15658-2)
Supplement: Supplementary file 4 — Description of Additional Supplementary Files [file 41467_2020_15658_MOESM4_ESM.docx]

**Description of Additional Supplementary Files**

File name: Supplementary Data 1
Description: Genes expressed in S9 petals. Genes expressed in S9 petals. Differential gene expression analysis was performed with DESeq2: Wald test, Benjamini and Hochberg’s correction.
