# Supplementary material for: The morphology, molecular development and ecological function of pseudonectaries on Nigella damascena (Ranunculaceae) petals
Source: Nat Commun. 2020 Apr 14;11:1777. doi: 10.1038/s41467-020-15658-2 (PMC7156421; doi:10.1038/s41467-020-15658-2)
Supplement: Supplementary file 1 — Peer Review [file 41467_2020_15658_MOESM1_ESM.pdf]

Reviewers' comments:

Reviewer #1 (Remarks to the Author):

In this manuscript, the authors describe a detailed and diverse study of the pseudonectaries of Nigella petals. These intriguing structures are glossy and seemingly attractive, but offer no reward to pollinators. Functionally similar structures have evolved several times independently in the Ranunculaceae, as well as other angiosperm lineages, but their molecular control has not been investigated in any system. The authors present data derived from SEM, histology, RNAseq, VIGS and ecological pollination studies. Overall, the data is excellent and clearly presented, but I do have some suggestions to improve the work.

Major revisions:

Lines 137-152: This section lacks some experiments that I might expect while also including material whose purpose is not entirely clear. I do not doubt that there is more cell proliferation in the area of the pseudonectaries but they also clearly have greater cell expansion. I might have expected the authors to perform in situ hybridization with a marker of cell division such as HIS4 or a cyclin to clearly demonstrate the longer persistence of cell division in this region. On the other hand, I am unclear as to the purpose of Fig. 3s. This appears to be purely an animation of an inferred developmental process. It is not based on live imaging or actual collection of data on cell numbers or size. The grids in the images do not appear to reflect actual relative cell sizes. There is no explanation of how this was generated in the materials and methods. While I find it a reasonable animation of the idea of pseudonectary growth, I'm not sure that it adds to the data presented in the rest of the figure.

Lines 215-218: I'm not sure the UV and bee vision images are that informative. In particular, I'm wondering if they could actually measure reflectance, as is often done in other papers on epidermal cell types.

Lines 277-279: I found this part a bit confusing. What do they mean by "…pseudonectaries function to distract desirable visitors from rewards intended for pollinators." Aren't pollinators desirable visitors? Do they mean undesirable?

Lines 302-331: I think the finding that NidaYAB5 is necessary for pseudonectary is a fascinating result. It is clear that it is more than just redeployment of abaxial identity simply to promote outgrowth since knockdown of NidaYAB5 eliminates all cell types associated with the pseudonectary. It is possible that the gene was initially ectopically expressed on the adaxial surface to promote outgrowth but it would appear that it now controls all aspects of pseudonectary development. Overall, I found this description a bit muddled and I think it can be improved.

Later in this section, I thought the statement that "if ectopic expression of YAB5 can explain the reason why pseudonectaries of one species are formed, it can explain the formation of pseudonectaries in other species." Do they just mean in Nigella? If so, that's fine but I think some evidence could be presented. Alternatively, are they suggesting that YAB5 has convergently been recruited to control pseudonectary formation in other Ranunculaceae or angiosperms? No evidence to support that has been presented, so this statement needs to be clarified.

Minor revisions:

Overall, the text is clear and well written but it could use revision by a technical editor. For instance, in line 53, the authors should say either "…, it is widely accepted that the nectary has…" or "…, it is widely accepted that nectaries have…". In line 77, I would suggest "…lack of suitable study systems…" In line 238, I would suggest "…it usually flew away after brief attempts."

Fig. 3 Legend: An explanation of panels 1-3 in relation to panels (i) and (m) should be added to the legend.

Reviewer #2 (Remarks to the Author):

Major claim: Liao et al. report that pseudonectaries in the flowers of Nigella damascena contribute to the attraction of pollinators and their orientation towards the nectar-secreting 'true' nectaries. The is conclusion is supported by experiments with plants that lacked pseudonectaries, which were created using VIGS to knock down a gene (NidaYABB5). This paper represents an outstanding contribution to the field. Hundreds of studies have reported ultrastructural and developmental aspects of nectaries, but fewer studies focused on pseudonectaries, and the potential function of these structures remained at the level of speculations.

Here, the authors start with a thorough 'classical' morphological and developmental study of the pseudonectaries. Then, they use transcriptomic analyses to discover genes of potential importance for the formation of pseudonectaries and they knock down a candidate gene. The corresponding phenotype lacks pseudonectaries, which clearly supports the key role of NidaYABB5 in their formation, and behavioural studies show that flowers without pseudonectaries received fewer visits by pollinators than wildtype plants and that pollinators spend less time on nectar probing in these flowers.

I have only one, major comment and some very minor remarks. I hope that these comments help the authors to optimize the potential impact of their work.

Major comment:
The pollination experiment represents the strongest but also the weakest part of the study. First, from the methods (line 393-394) it does not become clear where the experiment has been carried out (in the wild, in a botanical garden, or just in front of the greenhouse?). It does also not become clear how the "effective pollinators" (line 228) were discriminated from the nectar robbers (line 397 says "nectar rubbers", but I assume the authors refer to nectar robbers) and how the authors define "inefficient data" (line 397) which - apparently - were removed from the dataset. Due to these issues, I don't see how the authors tested the distraction hypothesis (line 288 states "the distraction scenario, however, was not supported in this study").

I strongly recommend to provide a much more detailed description of this part, particularly concerning the study site and the identification of efficient pollinators. Furthermore, the authors should state clearly whether Fig. 6h/i reports only visits of bees, or of all pollinators. They also should state in form of concrete numbers what a "substantial contribution of bumblebees and wasps" (line 228/229) means.

Minor remarks:
Perhaps I missed that detail, but what kind of gene is NidaYABB5? The authors characterise this gene as an "abaxial gene", but I think that some more explanations will be required to make its function comprehensible for a general readership.

The authors use several terms or expressions that do not meet current scientific standards. I already mentioned 'inefficient data' and 'substantial contribution'. Further examples:
Line 34: ..silence of it (the gene)
Line 68: …, pseudonectaries have not attracted enough attention
Line 96: …we carefully studied…
Line 98: … pseudonectaries are totally different

Referee: Martin Heil

Reviewer #3 (Remarks to the Author):

Pseudonectaries represent an extremely interesting flower feature that have received very little attention in the recent years. So, I warmly welcome this timely study representing the first attempt to investigate pseudonectaries with a multidisciplinary perspective. The authors employed a morphological, developmental, molecular and ecological investigation (including a manipulative experiment) for identifying the function of pseudonectaries in favoring the attraction of suitable pollinators and for marking the entrance of the nectar chamber, thereby guiding the pollinator to the hidden nectar. Results provided a strong support to the function of pseudonectaries in rewarding flowers in contrast to what has been found and discussed in carnivorous plants and deceptive flowers where pseudonectaries have a more obvious deceptive function.
I think the MS is very well written and data properly presented and discussed (even sometime Results anticipate some Discussions). Below I list a few minor comments/issues I suggest incorporating before the acceptance of this interesting paper

Introduction: I think, as pseudonectaries have been often found and described both in carnivorous plants and deceptive flowers (where their functions of deceiving the visiting insect is forthright) this should be mentioned and cited in the Introduction also because the conclusions of this MS point instead to a "positive" function of pseudonectaries in driving the pollinator to the hidden reward.

Line 140. A reference for the petal developmental stages is needed here.
Line 157: ….172 and 652 are specifically and preferably expressed in the pseudonectary-containing Part III …compared to what?? Something wrong in this sentence.

Line 184: ……Of the genes that are 185 up-regulated in Part III one… NidaYAB5 was not mentioned before.

Lines 257-268: All this descriptive section should be moved to the introduction

Lines 222-224. The description provided here is not very clear by looking at picture 6.

Lines 233-235. We do not know whether the TRV2-NidaYAB5-treated flowers also differ in scent emission so that other factors may reduce treated flower attractivity. A cautionary note should be applied.

Line 288. I simply do not think they TESTED this alternative hypothesis.

Line 300. I do not think this study provides evidence that pseudonectaries favor the attraction of more specific, preferred pollinators. And, in particular in this specific case, as the very generalist honey bee was the most frequent pollinator.

Lines 307-308. Is there any reference supporting this statement?

Line 394-396. Eighteen mocks and 18 TRV2-NidaYAB5-treated flowers with strong phenotypes were arrayed side by side (Fig. 6g). This does not correspond to what is showed in the Fig. 6g (where 16 flowers are showed)

Response to reviewers' comments

Reviewer #1

In this manuscript, the authors describe a detailed and diverse study of the pseudonectaries of *Nigella* petals. These intriguing structures are glossy and seemingly attractive, but offer no reward to pollinators. Functionally similar structures have evolved several times independently in the Ranunculaceae, as well as other angiosperm lineages, but their molecular control has not been investigated in any system. The authors present data derived from SEM, histology, RNAseq, VIGS and ecological pollination studies. Overall, the data is excellent and clearly presented, but I do have some suggestions to improve the work.

Lines 137-152: This section lacks some experiments that I might expect while also including material whose purpose is not entirely clear. I do not doubt that there is more cell proliferation in the area of the pseudonectaries but they also clearly have greater cell expansion. I might have expected the authors to perform in situ hybridization with a marker of cell division such as HIS4 or a cyclin to clearly demonstrate the longer persistence of cell division in this region. On the other hand, I am unclear as to the purpose of Fig. 3s. This appears to be purely an animation of an inferred developmental process. It is not based on live imaging or actual collection of data on cell numbers or size. The grids in the images do not appear to reflect actual relative cell sizes. There is no explanation of how this was generated in the materials and methods. While I find it a reasonable animation of the idea of pseudonectary growth, I'm not sure that it adds to the data presented in the rest of the figure.

Response: Thank you very much for the valuable suggestions. We actually do have expression data of *HIS4* (see Figure R1 in the next two pages) that show longer persistence of cell division in pseudonectaries. However, after careful evaluation, we decided not to include these results in this paper, for three reasons. First, as a widely used marker of cell division, *HIS4* itself has limits. Specifically, while the presence of its signal can indeed reflect the occurrence of cell division, the absence of the signal cannot exclude the possibility that the cell has just finished a round of cell division. Therefore, showing one or very few pictures is usually not enough. Second, petals of *Nigella damascena* are extremely complex and highly specialized in structure so that, in addition to pseudonectaries, there are several other characters that are also experiencing active cell division. The signal in pseudonectaries, therefore, can be easily obscured by those of other processes. Third, and more importantly, we are making measurement on the basis of the result, rather than the process, of cell division and cell expansion. Comparisons of the layers and size of cells in the pseudonectary and its flanking regions have clearly demonstrated the relative contributions of cell division and cell expansion to pseudonectary growth.

For Fig 3s, it is true that the pictures are purely animations of the inferred developmental process. It is not based on either live imaging or actual collection of data on cell numbers or size because we really don't have the data of the time that

each stage takes. The grids, however, only roughly reflect the relative size of the pseudonectaries caused by the collective effect of cell division and cell expansion. We believe that this information is helpful for understanding the process of pseudonectary growth, so we hope to keep it. Nevertheless, to better reflect our ideas, we have added explanations of how this was generated in materials and methods and revised Fig 3s to show the differences between the eight stages.

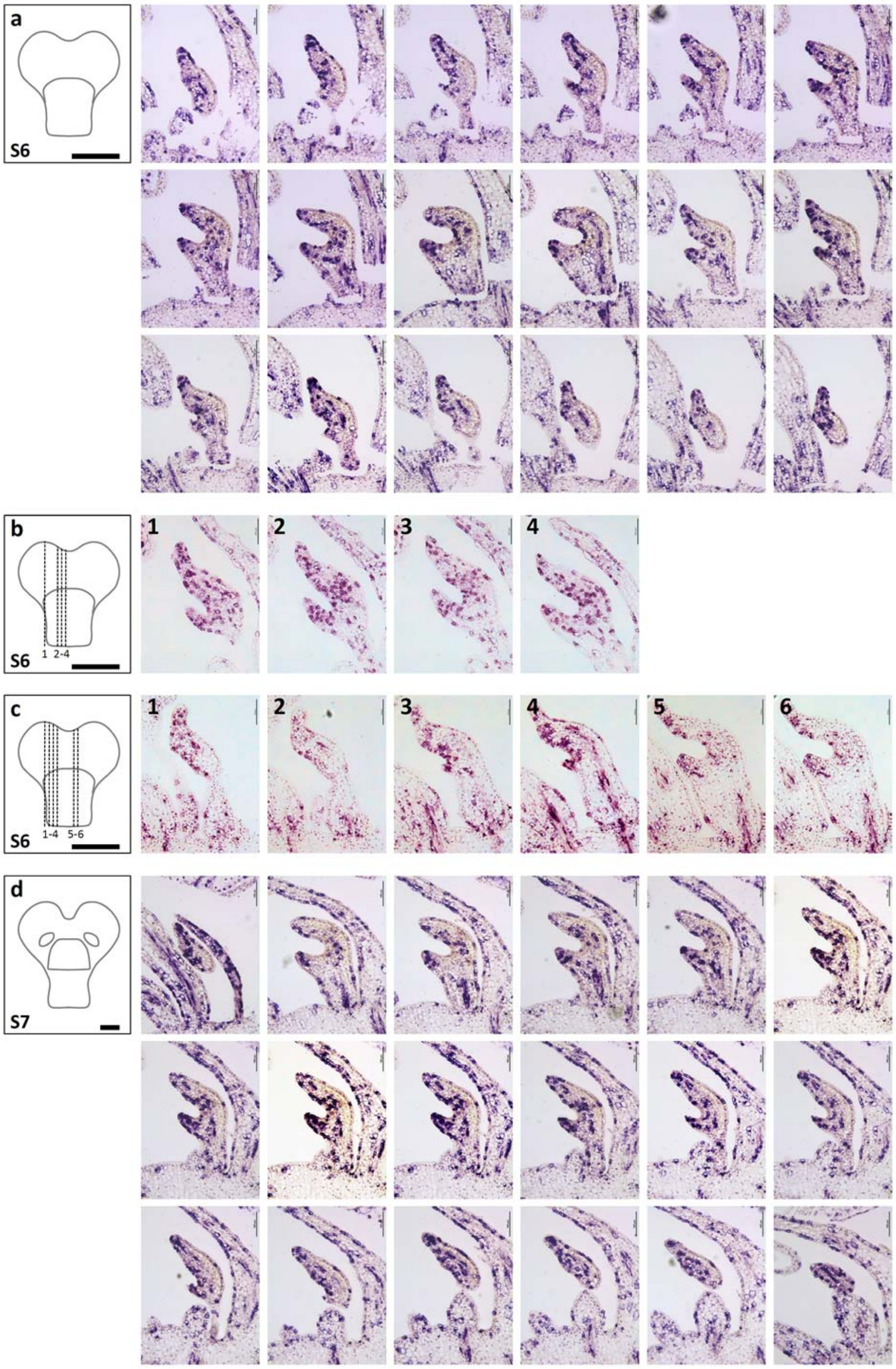

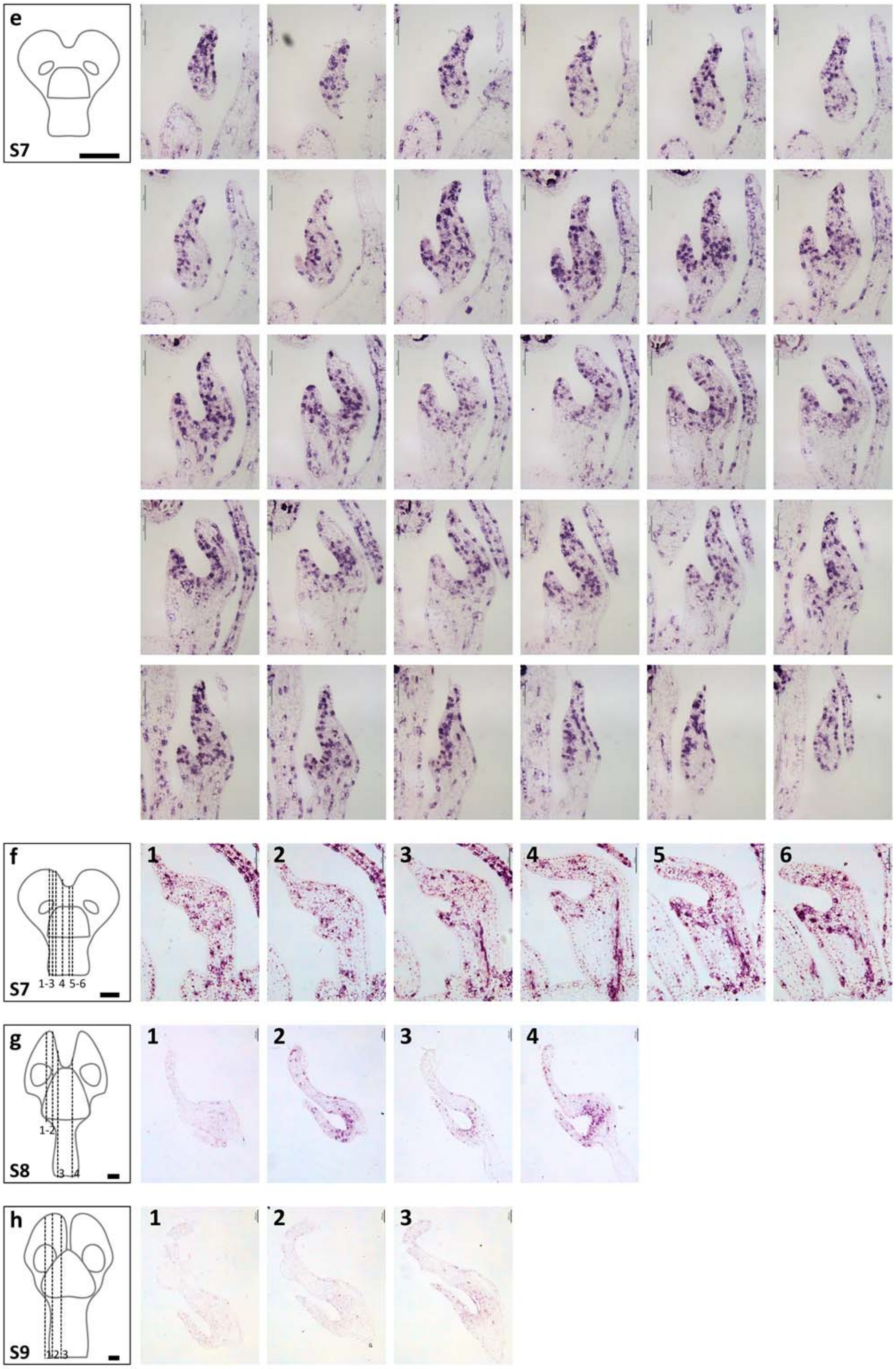

Figure R1. The expression pattern of the *HIS4* gene in petals of *Nigella damasena* at different developmental stages. Note that a, d and e each shows the complete sets of sections of a developing petal.

Lines 215-218: I'm not sure the UV and bee vision images are that informative. In particular, I'm wondering if they could actually measure reflectance, as is often done in other papers on epidermal cell types.

Response: Thanks for the suggestion. We agree that measuring reflectance would provide more direct evidence to the specialties of pseudonectaries. However, peseudonectaries of *N. damascena* are too tiny (about 700μm in diameter) to be measured by conventional instruments. In addition, we believe that the UV and bee vision images are already very informative because they clearly show that pseudonectaries are different from all other parts of the petal and because such kinds of strategy have been used in many studies (see Miller et al., 2011; Sheehan et al., 2016).

Miller, R., Owens, S. J., Rørslett, B. Plants and colour: flowers and pollination. ***Opt. Laser Technol.*** **43: 282-294 (2011).**

Sheehan, H. ***et al***. **MYB-FL controls gain and loss of floral UV absorbance, a key trait affecting pollinator preference and reproductive isolation.** ***Nat. Genet.*** **48: 159 (2016).**

Lines 277-279: I found this part a bit confusing. What do they mean by "… pseudonectaries function to distract desirable visitors from rewards intended for pollinators." Aren't pollinators desirable visitors? Do they mean undesirable?

Response: Sorry for the confusions. It is a typo. The word "desirable" has been changed to "**undesirable**".

Lines 302-331: I think the finding that *NidaYAB5* is necessary for pseudonectary is a fascinating result. It is clear that it is more than just redeployment of abaxial identity simply to promote outgrowth since knockdown of *NidaYAB5* eliminates all cell types associated with the pseudonectary. It is possible that the gene was initially ectopically expressed on the adaxial surface to promote outgrowth but it would appear that it now controls all aspects of pseudonectary development. Overall, I found this description a bit muddled and I think it can be improved.

Response: Thank you very much for the positive comments and valuable suggestions. It is true that the discussion here is not very clear. However, because we still don't know why knockdown of *NidaYAB5* not only led to complete loss of pseudonectaries but also eliminated all cell types associated with the pseudonectary, we cannot say too much about it. Nevertheless, following your suggestions, we have revised the corresponding sentences as follows:

In addition, we found that the abaxial gene *NidaYAB5* is a key regulator of pseudonectary formation; knockdown of it not only led to complete losses of pseudonectaries but also eliminated all cell types associated with pseudonectories. It is possible that the gene was initially ectopically expressed on the adaxial surface to promote outgrowth but then controls all aspects of pseudonectary development by regulating its downstream genes.

Later in this section, I thought the statement that "if ectopic expression of *YAB5* can explain the reason why pseudonectaries of one species are formed, it can explain the formation of pseudonectaries in other species." Do they just mean in *Nigella*? If so, that's fine but I think some evidence could be presented. Alternatively, are they suggesting that *YAB5* has convergently been recruited to control pseudonectary formation in other Ranunculaceae or angiosperms? No evidence to support that has been presented, so this statement needs to be clarified.

Response: Yes, we mean in *Nigella*, and this has been clarified in the text. Up to now, we don't have any idea about the situation in other Ranunculaceae or angiosperms.

Overall, the text is clear and well written but it could use revision by a technical editor. For instance, in line 53, the authors should say either "…, it is widely accepted that the nectary has…" or "…, it is widely accepted that nectaries have…".

Response: Thanks for the positive comments and valuable suggestions. We have checked the manuscript and made some revisions. In Line 53, "nectary" has been changed to "**the nectary**".

In line 77, I would suggest "…lack of suitable study systems…"

Response: Thanks. Corrected.

In line 238, I would suggest "…it usually flew away after brief attempts."

Response: Thanks. Corrected.

Fig. 3 Legend: An explanation of panels 1-3 in relation to panels (i) and (m) should be added to the legend.

Response: Based on your description, we guess that you are talking about Fig. 5 rather than Fig. 3. To explain the relationships between panels 1-3 and panels (i) and (m), we have added one sentence into the legend of Fig. 5, which now reads:

Panels 1-3 next to (i) and (m) indicate the corresponding regions on the surface of the mock and TRV2-*NidaYAB5*-treated petals.

Reviewer #2

Major claim: Liao et al. report that pseudonectaries in the flowers of *Nigella damascena* contribute to the attraction of pollinators and their orientation towards the nectar-secreting 'true' nectaries. The is conclusion is supported by experiments with plants that lacked pseudonectaries, which were created using VIGS to knock down a gene (*NidaYABB5*). This paper represents an outstanding contribution to the field. Hundreds of studies have reported ultrastructural and developmental aspects of

nectaries, but fewer studies focused on pseudonectaries, and the potential function of these structures remained at the level of speculations.

Here, the authors start with a thorough 'classical' morphological and developmental study of the pseudonectaries. Then, they use transcriptomic analyses to discover genes of potential importance for the formation of pseudonectaries and they knock down a candidate gene. The corresponding phenotype lacks pseudonectaries, which clearly supports the key role of *NidaYABB5* in their formation, and behavioural studies show that flowers without pseudonectaries received fewer visits by pollinators than wildtype plants and that pollinators spend less time on nectar probing in these flowers.

I have only one, major comment and some very minor remarks. I hope that these comments help the authors to optimize the potential impact of their work.

The pollination experiment represents the strongest but also the weakest part of the study. First, from the methods (line 393-394) it does not become clear where the experiment has been carried out (in the wild, in a botanical garden, or just in front of the greenhouse?).

It does also not become clear how the "effective pollinators" (line 228) were discriminated from the nectar robbers (line 397 says "nectar rubbers", but I assume the authors refer to nectar robbers) and how the authors define "inefficient data" (line 397) which - apparently - were removed from the dataset. Due to these issues, I don't see how the authors tested the distraction hypothesis (line 288 states "the distraction scenario, however, was not supported in this study").

I strongly recommend to provide a much more detailed description of this part, particularly concerning the study site and the identification of efficient pollinators.

Response: Thank you very much for the valuable comments and remarks. The pollination experiment was carried out in Institute of Apicultural Research, Chinese Academy of Agricultural Sciences. The institute, in turn, is located within the Beijing Botanical Garden, so that various kinds of potential visitors and pollinators are available. For the experiment, plants with flowers with and without pseudonectaries were arrayed side by side (Fig. 6k) on an open lawn from 9:00 am to 17:00 pm in four sunny and calm days of July, 2017. To reflect this, we have revised the corresponding parts and added descriptions of effective pollinators, nectar robbers and efficient data. The revised sentences now read:

Pollination studies were carried out in an open area of the Institute of Apicultural Research, Chinese Academy of Agricultural Sciences from 9:00 am to 17:00 pm in four sunny and calm days of July, 2017.Visitors being able to successfully transfer pollens from pollen sac to stigma were regarded as pollinators, while those that suck nectar but don't transfer pollens were regarded as nectar robbers. The most effective pollinators are the pollinators that have the highest frequency of visitation and longest time of probing. For the experiment, flowers with and without pseudonectaries (i.e., 18 mock flowers and 18

TRV2-*NidaYAB5*-treated flowers with strong phenotypic changes) were arrayed side by side (Fig. 6k). For each kind of potential pollinators, both the visiting frequency and probing time of pollinators were recorded. After filtering inefficient data (i.e., visitation of nectar robbers), significance evaluation of the two pollination parameters (*P* value) were determined by using non-parametric Wilcoxon signed rank test.

Also, it is true that our data cannot test the distraction hypothesis. The sentence, therefore, has been changed to: "**The distraction scenario, however, cannot be supported or rejected in this study.**"

Furthermore, the authors should state clearly whether Fig. 6h/i reports only visits of bees, or of all pollinators. They also should state in form of concrete numbers what a "substantial contribution of bumblebees and wasps" (line 228/229) means.

Response: Thank you for the suggestion. The Fig. 6l/m reports only visits of honey bees, the most effective pollinator. To make it clear, the statement of Fig. 6 l/m in the figure legend have been changed to **"l-m, Comparisons of the visiting frequency (l) and probing time (m) of honey bees, the most effective pollinators, on the mock and TRV2-*NidaYAB5*-treated flowers."** Furthermore, we have changed the statement at lines 229-230 to "**the contribution of bumblebees (*Bombus lucorum*) and wasps (*Polistes dominulus*) were also substantial (about 10% of the recorded times of visitations)**".

Perhaps I missed that detail, but what kind of gene is *NidaYABB5*? The authors characterise this gene as an "abaxial gene", but I think that some more explanations will be required to make its function comprehensible for a general readership.

Response: Thank you for the suggestion. *NidaYAB5* attracted our special attention because it is the only abaxial/adaxial gene that are up-regulated in Part III and because ectopic expression of adaxial/abaxial genes have been shown to play key roles in the formation of outgrowths on leaf-like structures. The expression pattern of *NidaYAB5* has been shown in Fig. 4 e,f,h. To reflect this, we have cited the figures and reworded this part a little bit, which now reads:

Of the genes that are up-regulated in Part III, one (i.e., *NidaYAB5*; Fig. 4e, f, h) attracted our special attention because it is the ortholog of a known abaxial gene (i.e., *YABBY5*) [24] and because ectopic expression of adaxial/abaxial genes have been shown to play key roles in the formation of outgrowths on leaf-like structures [48-52].

The authors use several terms or expressions that do not meet current scientific standards. I already mentioned 'inefficient data' and 'substantial contribution'. Further examples:
Line 34: … silence of it (the gene)
Line 68: …, pseudonectaries have not attracted enough attention
Line 96: … we carefully studied…
Line 98: … pseudonectaries are totally different

Response: Thanks for the suggestions!
Line 34: "silence" has been replaced by "**knockdown**".
Line 68: "have not attracted enough attention" has been changed to "**have not yet attracted sufficient attention**".
Line 96: "we carefully studied" have been changed to "**we investigated**".
Line 98: "totally" has been changed to "**quite**".

Reviewer #3

Pseudonectaries represent an extremely interesting flower feature that have received very little attention in the recent years. So, I warmly welcome this timely study representing the first attempt to investigate pseudonectaries with a multidisciplinary perspective. The authors employed a morphological, developmental, molecular and ecological investigation (including a manipulative experiment) for identifying the function of pseudonectaries in favoring the attraction of suitable pollinators and for marking the entrance of the nectar chamber, thereby guiding the pollinator to the hidden nectar. Results provided a strong support to the function of pseudonectaries in rewarding flowers in contrast to what has been found and discussed in carnivorous plants and deceptive flowers where pseudonectaries have a more obvious deceptive function.

I think the MS is very well written and data properly presented and discussed (even sometime Results anticipate some Discussions). Below I list a few minor comments/issues I suggest incorporating before the acceptance of this interesting paper.

Introduction: I think, as pseudonectaries have been often found and described both in carnivorous plants and deceptive flowers (where their functions of deceiving the visiting insect is forthright) this should be mentioned and cited in the Introduction also because the conclusions of this MS point instead to a "positive" function of pseudonectaries in driving the pollinator to the hidden reward.

Response: Thanks for the valuable suggestions. We have added the following sentence to reflect this: **Pseudonectaries of carnivorous plants (e.g., *Cephalotous follicularis*) and deceptive flowers (e. g., *Ophrys muscifera*) also function to deceive the visiting insects[36,37].**

Matthews, M. L. & Endress, P. K. J. B. J. o. t. L. S. Comparative floral structure and systematics in Oxalidales (Oxalidaceae, Connaraceae, Brunelliaceae, Cephalotaceae, Cunoniaceae, Elaeocarpaceae, Tremandraceae). *Bot. J. Lin. Soc.* 140, 321-381 (2002).
Waser, N. M., Ollerton, J. & Erhardt, A. J. J. o. P. E. Typology in pollination biology: lessons from an historical critique. *J. Pollinat. Ecol.* 3 (2011).

Line 140: A reference for the petal developmental stages is needed here.

Response: Thank you for the suggestion. A reference shown below for the petal developmental stages has been cited after "**petal development** [28]".

Yao, X. *et al*. The making of elaborate petals in *Nigella* through developmental repatterning. *New Phytol.* 223, 385-396 (2019).

Line 157: ….172 and 652 are specifically and preferably expressed in the pseudonectary-containing Part III …compared to what?? Something wrong in this sentence.

Response: Thank you for the suggestion. We have revised the sentence at line 157 to **"Of the 21,223 genes that are expressed in the petals of this stage, 172 and 652 are specifically and preferably expressed in the pseudonectary-containing Part III as compared to the other three parts (Fig. 4c,d, Supplementary Dataset 1), respectively, suggestive of the uniqueness of this part."**

Line 184: ……Of the genes that are 185 up-regulated in Part III one… *NidaYAB5* was not mentioned before.

Response: Thank you for the suggestion. The gene has actually been shown in Fig. 4 e, f, h. Nevertheless, we have revised the sentence at lines 184 -185 to:

Of the genes that are up-regulated in Part III, one (i.e., *NidaYAB5*; Fig. 4e, f, h) attracted our special attention because it is the ortholog of a known abaxial gene (i.e., *YABBY5*) [24] and because ectopic expression of adaxial/abaxial genes have been shown to play key roles in the formation of outgrowths on leaf-like structures [48-52].

Lines 257-268: All this descriptive section should be moved to the introduction.

Response: Thanks for the suggestion. This has been moved to the introduction.

Lines 222-224: The description provided here is not very clear by looking at picture 6.

Response: Thanks for the suggestion. To make our description clear, we have added schematic diagrams in **Fig. 6 (g-j; see below)** and some information in figure legend**.**

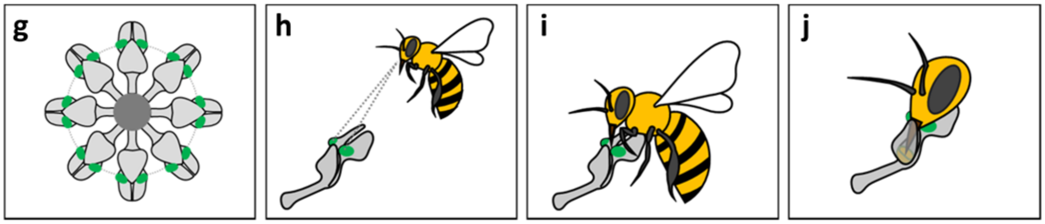

Lines 233-235: We do not know whether the TRV2-*NidaYAB5*-treated flowers also differ in scent emission so that other factors may reduce treated flower attractivity. A cautionary note should be applied.

Response: Yes, you are right. We therefore have added "tend to" in front of "suggest" to reflect our cautions. The sentence now reads:

Taken together, these results tend to suggest that pseudonectaries can not only attract suitable pollinators but also mark the entrance of the nectar chamber, thereby guiding their visitation.

Line 288: I simply do not think they TESTED this alternative hypothesis.

Response: Our results truly cannot test the alternative hypothesis at line 288. The sentence, therefore, has been changed to: "**The distraction scenario, however, cannot be supported or rejected in this study.**"

Line 300: I do not think this study provides evidence that pseudonectaries favor the attraction of more specific, preferred pollinators. And, in particular in this specific case, as the very generalist honey bee was the most frequent pollinator.

Response: Yes, honey bees are generalists. However, this means that <u>honey bees</u> can visit and pollinate for many kinds of flowers; this does not mean that <u>flowers</u> can attract and accept many kinds of visitors and pollinators. For *Nigella damascena*, honey bees are one of the very few types of effective (and preferred) pollinators.

Lines 307-308: Is there any reference supporting this statement?

Response: Yes. We have cited two references at lines 307-308:

Waters, M. T. *et al*. GLK transcription factors coordinate expression of the photosynthetic apparatus in *Arabidopsis*. *Plant Cell* 21, 1109-1128 (2009);
Aharoni, A. *et al*. The SHINE clade of AP2 domain transcription factors activates wax biosynthesis, alters cuticle properties, and confers drought tolerance when overexpressed in *Arabidopsis*. *Plant Cell* 16, 2463-2480 (2004).

Line 394-396: Eighteen mocks and 18 TRV2-*NidaYAB5*-treated flowers with strong phenotypes were arrayed side by side (Fig. 6g). This does not correspond to what is showed in the Fig. 6g (where 16 flowers are showed).

Response: Thanks for the suggestion. We have revised Fig. 6k to reflect the real positions of the 18 mocks and 18 *TRV2-NidaYAB5*-treated flowers.

Reviewer #1 (Remarks to the Author):

I'm perfectly satisfied with the revisions.

Reviewer #2 (Remarks to the Author):

Thank you very much for your replies to my comments. I am happy to see that my comments were helpful and I am fully satisfied with the changes made to the manuscript. I have no further suggestions to make. Congratulations on a very nice study!
Martin Heil

Reviewer #3 (Remarks to the Author):

I am happy with this revised version of this MS. The authors have properly acknowledged the largest part of comments and adequately addressed to the very few ones they have not included
